# Endoscopic Ultrasound-Guided Glue Embolization for Left Gastric Artery Pseudoaneurysm Presenting as Hemosuccus Pancreaticus After Failed Radiological Intervention

**DOI:** 10.14309/crj.0000000000001271

**Published:** 2024-02-16

**Authors:** Praveer Rai, Pankaj Kumar, Prabhat Kumar Verma, Kartik Agarwal, Anil Kumar Singh

**Affiliations:** 1 Department of Gastroenterology, Sanjay Gandhi Postgraduate Institute of Medical Sciences, Lucknow, Uttar Pradesh, India; 2 Department of Radiodiagnosis, Sanjay Gandhi Postgraduate Institute of Medical Sciences, Lucknow, Uttar Pradesh, India

**Keywords:** hemosuccus pancreaticus, pseudoaneurysm, left gastric artery, endoscopic ultrasound, glue embolization

## Abstract

Hemosuccus pancreaticus is characterized by intermittent bleeding from the ampulla of Vater due to the rupture of a pseudoaneurysm. There are significant diagnostic and therapeutic challenges associated with this rare and potentially life-threatening condition. We present a rare case in an 18-year-old man who presented with recurrent episodes of hematemesis and melena due to hemosuccus pancreaticus as a result of a left gastric artery pseudoaneurysm. Initial radiological angioembolization failed because of median arcuate ligament syndrome, and endoscopic ultrasound-guided glue embolization was successfully performed. This case further reinforces the importance of endoscopic ultrasound-guided therapy in the management of pseudoaneurysm after failed radiological treatment.

## INTRODUCTION

Hemosuccus pancreaticus is generally characterized by intermittent upper gastrointestinal (GI) bleeding from the ampulla of Vater. There is a rare and challenging condition that occurs after pseudoaneurysm rupture. Splenic artery pseudoaneurysm (37.9%) is the most common source of bleeding, followed by gastroduodenal artery aneurysm (21.8%), superior mesenteric artery aneurysm, and unnamed cystic wall vessels.^1^ Pseudoaneurysms of the left gastric artery are relatively uncommon, accounting for less than 1% of all cases. Most commonly, hemosuccus pancreaticus is associated with chronic pancreatitis (75.8%), acute necrotizing pancreatitis (12.6%), and recurrent acute pancreatitis (4.6%).^1^ We report a case of left gastric artery pseudoaneurysm in a patient with recurrent hematemesis and melena. Despite unsuccessful radiological angioembolization because of median arcuate ligament syndrome, we effectively managed the patient using endoscopic ultrasound-guided glue embolization, demonstrating a minimally invasive and successful treatment approach.^2^

## CASE REPORT

An 18-year-old man presented with 1-day pancreatic-type abdominal pain, vomiting, and paralytic ileus in September 2022, requiring hospitalization for 5 days, and was diagnosed with acute pancreatitis. Subsequently, he experienced recurring mild dull aching pain in the epigastrium every 15–20 days, which was manageable with oral analgesics and did not require hospitalization. The patient experienced hematemesis and melena in January 2023 without requiring packed red blood cells (PRBCs). In June 2023, he had a 2-day history of abdominal pain, followed by 2 bouts of hematemesis (approximately 200–300 mL each) and melena, leading to a significant drop in hemoglobin levels, requiring 2 units of PRBC transfusion. The patient presented to our tertiary care center 1 month later with hematemesis and melena. The patient was pale on examination and had a pulse rate of 110 beats per minute and blood pressure of 90/60 mm Hg. There was a significant drop in hemoglobin (from 12 to 7 g/dL). An upper GI endoscopy revealed fresh blood oozing from the ampulla of Vater, consistent with a hemosuccus pancreaticus (Video 1).

Video 1 Upper gastrointestinal endoscopy showing hemosuccus pancreaticus.

Computed tomography (CT) angiography was performed to identify the source of the bleeding, which revealed a mild peripancreatic fat stranding with a heterogeneous pancreas, normal bulk, and no calcification. The pancreatic duct was not dilated. A collection measuring 4.4 × 3.5 × 3.2 cm with an internal hyperdense content (60 HU), suggestive of bleeding, was observed in the peripancreatic region, superior to the pancreatic body. In addition, a 7.7 mm contrast-filled outpouching, likely a pseudoaneurysm, was also observed; hence, acute pancreatitis with peripancreatic fluid collection along with a left gastric artery pseudoaneurysm was diagnosed.

### Interventions and management

Given the patient's unstable hemodynamic status and recurrent bleeding, prompt intervention was necessary. Digital subtraction angiography was performed through the right common femoral artery, and angiography of the celiac trunk and superior mesenteric artery revealed collateral flow from the superior mesenteric artery to the celiac trunk branches, with evidence of induration and partial compression of the celiac trunk, indicative of median arcuate ligament syndrome. The angiogram also revealed a small, left gastric artery (LGA) partially thrombosed pseudoaneurysm. Despite repeated attempts to cannulate the LGA, there was no success because of the median arcuate ligament syndrome, which prevented endovascular treatment. The procedure was concluded with the removal of the right groin access sheath and successful local hemostasis.

**Figure 1.**
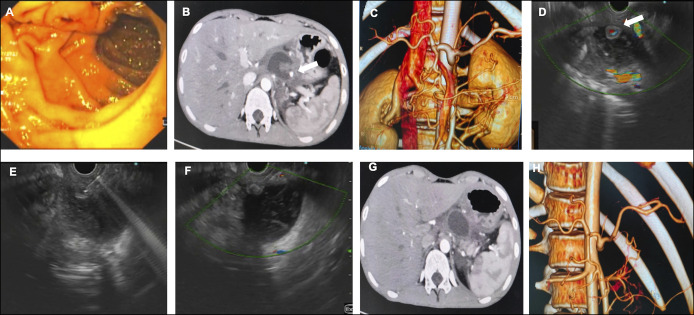
Image sequence illustrating the diagnosis and treatment process of a pseudoaneurysm. Blood oozing through the papilla was observed in the upper GI endoscopy image (A). The CT image (B) shows a small pseudoaneurysm in the LGA. The coronal section (C) and reconstructed image (D) provide more detailed views. The aneurysm was completely obliterated on the CT scan image (E), which is also shown in the reconstructed image (F). CT, computed tomography; GI, gastrointestinal; LGA, left gastric artery.

The patient experienced 1 bout of hematemesis (approximately 100 mL of fresh blood) and subsequent melena for 3 days, after the procedure, accompanied by a 1 g drop in hemoglobin, after which 1 unit of PRBCs was transfused. Based on our prior experience with EUS-guided therapy for pseudoaneurysms, we decided to proceed with EUS-guided intervention.^2–4^ A linear echoendoscope (GF UCT 180; Olympus, Tokyo, Japan) positioned in the stomach detected a 5 mm pseudoaneurysm originating from the LGA branch. The pseudoaneurysm was punctured with a 22-gauge EUS fine-needle aspiration needle (Expect; Boston Scientific, Marlborough, MA) using color Doppler to avoid intervening blood vessels (Figure 1, Video 1). A needle stylet was withdrawn after puncturing the aneurysm, and 0.5 mL of glue (n-butyl-2-cyanoacrylate) was injected. After glue injection, the pseudoaneurysm was completely occluded, and a Doppler examination revealed no flow.

### Follow-up and outcome

After EUS-guided glue embolization, the patient's hemoglobin levels stabilized and he remained hemodynamically stable. During the hospitalization, there was no further GI bleeding. Repeat EUS after 1 week confirmed complete occlusion of the pseudoaneurysm. The patient remained symptom-free during the three-month follow-up period after discharge. The pseudoaneurysm was completely obliterated by repeat CT angiography of the abdomen.

## DISCUSSION

Hemosuccus pancreaticus is an uncommon cause of upper GI bleeding, mostly affecting men. The typical presentation includes epigastric pain with back radiation accompanied by intermittent GI bleeding. Raised serum amylase levels were also observed. Most commonly, the splenic artery is implicated, but other arteries including the gastroduodenal, pancreaticoduodenal, and left gastric arteries may also be involved.^5^ In rare cases, hemosuccus pancreaticus may result from pancreatolithiasis or pancreatic pseudocyst.^6,7^ Aneurysms can either be pseudoaneurysms or true aneurysms. Both are associated with acute or chronic pancreatitis. Pseudoaneurysms carry a 37% risk of rupture and 90% mortality rate, in the absence of treatment.^8^ CT mesenteric angiography is typically used to detect the hemosuccus pancreaticus. In 30% of cases, upper GI endoscopy can detect bleeding through the papilla, but hemorrhage tends to be intermittent, making diagnosis difficult.^7^ Interventional radiology treatment modalities, particularly endovascular coiling by angiography, are considered initial therapeutic approaches. In cases where radiological treatment proves ineffective or the source of bleeding remains unidentified, surgical intervention is pursued, involving aneurysm resection, splenectomy for distal aneurysms, or resection of the pancreatic head or tail.

LGA pseudoaneurysm presenting as a hemosuccus pancreaticus is a rare clinical entity that requires prompt diagnosis and intervention. In the literature, only 2 cases of LGA pseudoaneurysm presenting as a hemosuccus pancreaticus have been reported, both with a history of chronic pancreatitis. The first case was managed with emergency surgery to excision and ligation of the pseudoaneurysm sac while the second case was treated with radioembolization.^9,10^

Our case of a left gastric artery pseudoaneurysm presenting as a hemosuccus pancreaticus was managed with EUS-guided therapy. This further emphasizes that EUS-guided glue embolization is an effective and minimally invasive treatment option for the management of pseudoaneurysm. We recommend a lower threshold for EUS-guided therapy for patients with similar clinical presentations. Further studies are required to explore the long-term outcomes and efficacy of this treatment modality.

## DISCLOSURES

Author contributions: All authors contributed equally to this manuscript. P. Rai is the article guarantor.

Financial disclosure: None to report.

Informed consent was obtained for this case report.
